# Resonance-based sparse adaptive variational mode decomposition and its application to the feature extraction of planetary gearboxes

**DOI:** 10.1371/journal.pone.0231540

**Published:** 2020-04-13

**Authors:** Jing Zhu, Aidong Deng, Jing Li, Minqiang Deng, Wenqing Sun, Qiang Cheng, Yang Liu

**Affiliations:** 1 National Engineering Research Center of Turbo-Generator Vibration, School of Energy and Environment, Southeast University, Nanjing, China; 2 School of Information Engineering, Nanjing Audit University, Nanjing, China; National Huaqiao University, CHINA

## Abstract

Due to the assumption that the VMD technique is essentially a set of adaptive Wiener filter banks and its performance depends to a large extent on the preset parameter K (the number of decomposition). A new method named resonance-based sparse adaptive variational mode decomposition (RSAVMD) is proposed for the decomposition of planetary gearbox vibration signals. Tunable Q-Factor Wavelet Transform (TQWT) and morphological component analysis (MCA) are introduced to decompose the original signal into high and low resonance components. High resonance components containing planetary gearbox signals are screened for analysis. At the same time, Quality factor is used to select the number of Variational mode decomposition (VMD) adaptively. This method was applied in fault diagnosis of planetary gearbox. Compared with VMD, RASVMD could extract fault characteristic frequency of planetary gearbox accurately, but VMD lost part of fault information, showing the superiority of RSAVMD. Simultaneously, the selection method of VMD decomposition number in literature was cited, and it was found that the decomposition number selected by the method in this paper was more accurate.

## Introduction

Planetary gearboxes have large power transmission capacity in a compact structure, small volume, light weight, big transmission ratio and high transmission efficiency. Therefore they occupy the main position in the field of industrial production and engineering, such as wind turbines, helicopters, construction machines, and other types of transmission systems. Most planetary gear boxes work in some severe operation condition with low speed and alternating loads, which are particularly prone to the failure [1]. Diagnosing the faults and research on the evaluation of gearbox conditions are helpful to improve the safety, reliability and stability of gearbox operation.

The transmission by planetary gear sets has some unique characteristics: Firstly, each planet gear is meshing with the ring and the sun gears simultaneously; Secondly, the vibration transfer paths between the gears meshing positions and the mounted sensor time-varying because the epicyclic motion of planet gears revolve around the sun gear. Thirdly, the structure of a planetary set is complicated. As a result, the observed vibration signals rich in various components and modulations, which make the identification of the tooth fault related modulation sidebands more difficult for the planetary gear sets, i.e. A lot of components appear in the spectrum [2–5]. All these factors make the characteristic frequency of fault difficult to extract.

In view of nonlinearity and nonstationarity of the planetary gearbox fault signal under the strong background noise, the method of fault feature extraction based on time-frequency analysis has been widely developed [6–7]. Zhipeng Feng exploited the capability of surrogate test technique in recognizing true signal components and proposed thereby an adaptive iterative generalized demodulation (AIGD) [8]. Minqiang Deng proposed a new optimized Fourier spectrum decomposition method, termed bandwidth Fourier decomposition (BFD) [9].Many optimization algorithms are also proposed to solve related problems [10–11]. Among the methods used commonly in fault diagnosis, the time synchronous averaging (TSA), narrowband demodulation are not applicable for the localized gear teeth faults detection in planetary gear sets directly. The time-frequency window of the short-time Fourier transform is fixed, Time and frequency feature could be constructed. Wigner-ville is one of the useful time frequency analysis method to express time serial signal variation. However, Wigner-ville has cross-term problems [12]. The instantaneous frequency of the same component of Hilbert transform varies greatly at different times, and its accuracy needs to be improved; Among them, EMD and EEMD [13] are widely used in fault diagnosis due to their advantages of high time-frequency resolution and good self-adaptation, but there are problems such as mode mixing and endpoint effect inevitably.

In response to the above problems, Dragomiretskiy and Zosso [14] proposed variational mode decomposition (VMD) based on traditional Wiener filtering. VMD can control the phenomenon of mode mixing effectively and restrain the influence of sampling effect by controlling the convergence condition. In recent years, it has become a hot spot in various applications of science [15–18]. Lahmiri made a comparative study of 11 widely used techniques in the literature, it is found that LCDP in combination with VMD performs the best and that VMD is faster than EMD [19]. A retinal image is processed with variational mode decomposition (VMD) to detect hemorrhages in the retina by Salim [20]. Lahmiri explored the usefulness of fractal descriptors estimated in multi-resolution domains to characterize biomedical digital image texture. The results demonstrate that fractal descriptors estimated in VMD domain outperform those estimated in DWT and EMD domains; and also those directly estimated from the original image [21].However, it still has the following two shortcomings:

Firstly, since VMD is a multi-adaptive Wiener filter group essentially. The noise interference in the decomposition process is much less than that of EMD, but the Wiener filter works well for stationary random signals. For the non-stationary signal of the planetary gearbox, the decomposition of the Wiener filter is less robust. Furthermore, it is worth noting that VMD works in the frequency domain based on the assumption of the narrowband property of the signal mode. Once the frequency spectrum shows the wideband characteristics imposed by the frequency modulation phenomenon, the VMD ceases to be effective.

To overcome this limitation, many demodulation techniques are used to remove the effects of modulation to get the required narrowband demodulated signal [22–24]. Nevertheless, these approaches ask for an valid method to reckon the phase function accurately.

Secondly, When performing variational mode decomposition on a signal, the total number of extracted BLIMFs (set as K)is necessary to be preset artificially, which restricts the practicability of VMD, because the error of K relative to the actual value would cause mode discard or mode mixing. Xiao et al proposed an approach which select the IMF with largest kurtosis and appropriate correlation coefficients simultaneously. However, Kurtosis measures the impact of a signal and is not suitable for the selection of wideband signal.

This paper makes some possible extensions to VMD to effectively decompose planetary fault signals. The theoretical basis of the proposed method is that wideband signals with similar center frequencies and overlapping components can be separated, not on the basis of frequency or scale as provided by Fourier and wavelet transforms, but on the basis of resonance. As mentioned above, the essence of VMD is multiple adaptive wiener filter banks, but the use of filter must be under certain conditions: only frequency domain, that mean the effective signals and noise can be separated only when there is no overlap between the spectrum of them. However, the signal that we encounter in reality is not like this, the spectrum of noise and interference signals even distribute throughout the frequency domain. From the perspective of resonance properties, Resonance-based sparse decomposition considers the factors of center frequency and frequency bandwidth comprehensively, and overcomes the shortcoming of traditional filtering method that center frequency is similar and frequency band overlaps. Signal components with similar center frequency but different resonance properties can be separated effectively, and wideband signal which is not to do with planetary gear fault signals can be eliminated, meets the assumption that the signal modes in VMD have narrow-band characteristics.

In this paper, the proposed method is named resonance-based sparse adaptive variational mode decomposition (RSAVMD),which select the parameter K adptively at the same time. In addition, the validity of the method is verified by the experimental signals of the planetary gearbox. The results show that RSAVMD can effectively remove noise while separating very weak signal components, and its effect is better than VMD.

Hereafter, the paper is organized as follows: Firstly, this paper provides a brief overview on the principle of the VMD and Tunable Q-Factor Wavelet Transform. Secondly, based on the above theories and methods, the implementation steps of the method RSAVMD are presented. Thirdly, this paper shows that the RSAVMD can be successfully applied to extract fault characteristics of the experimental vibration signals in the planetary gear `box. Finally, some conclusions are drawed.

## Materials

In this section, a few concepts and methodologies that establish the foundation of the proposed method are briefly reviewed.

### 1. Resonance-based sparse decomposition

The magnitude of Q reflects the resonance degree of the signal. Quality factor (Q) is defined as:

$$Q=\frac{f_{c}}{BW}$$ (1)

**Fig 1:**
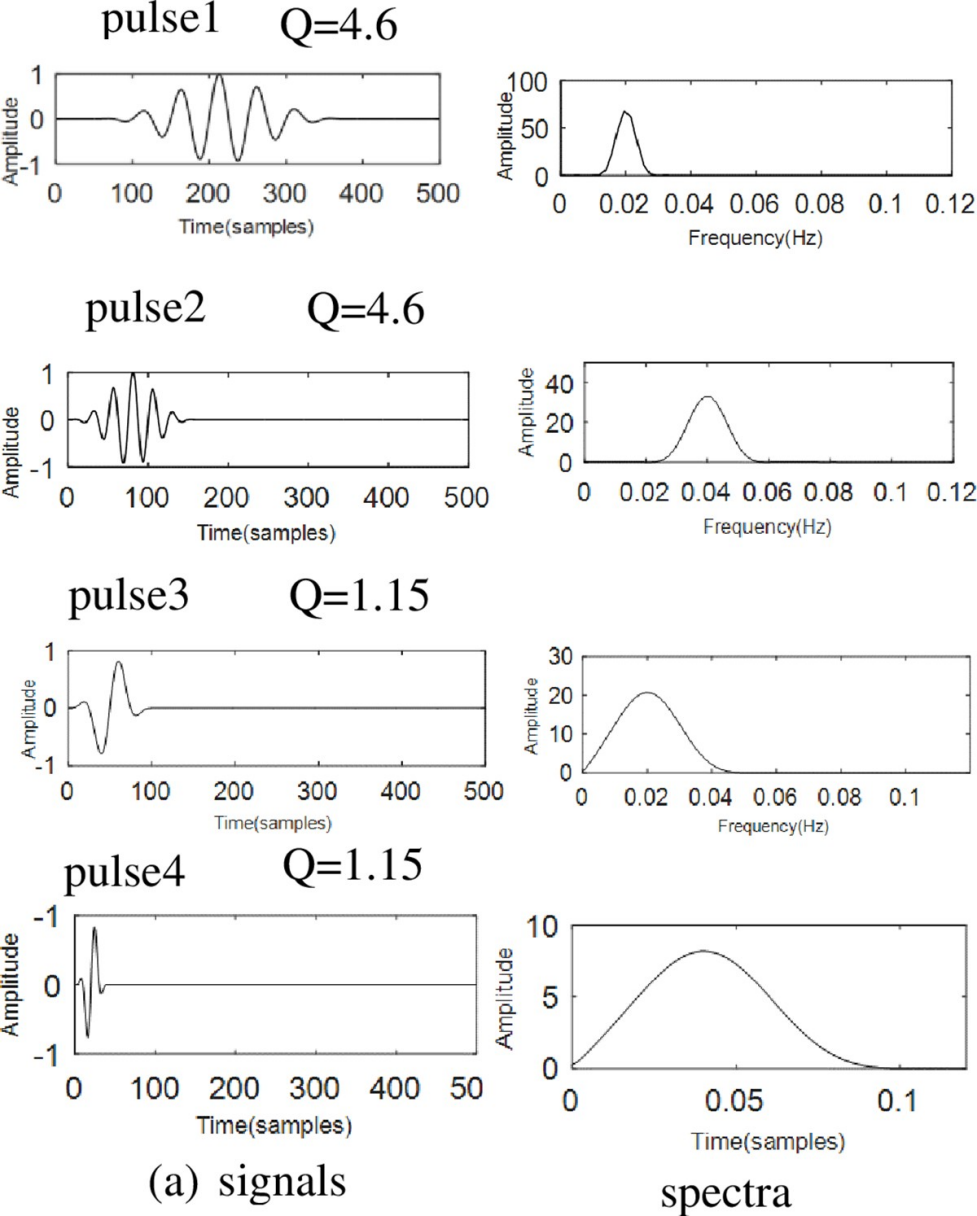
Signals and its spectra with different Q. The figure shows four impulse signals whose Q are equal to 4.6. Plus1 and Plus2 are high resonance components which have high impact frequency. It's a broadband signal in the frequency domain. Similarly, Plus3 and Plus4 are low resonance components which are narrow-band signal.

Where, *f*_*c*_ is the central frequency of the signal, and *BW* is the bandwidth of the signal. As shown in the Fig 1, The resonance of an independent pulse signal could be quantified by its Q-factor.

The oscillations of high-resonance pulses are more sustained, the pulse1 is a low frequency signal and the pulse2 is a high frequency signal, but they have same resonance property. Therefore, the resonance property and frequency of a signal are not related.

**Fig 2:**
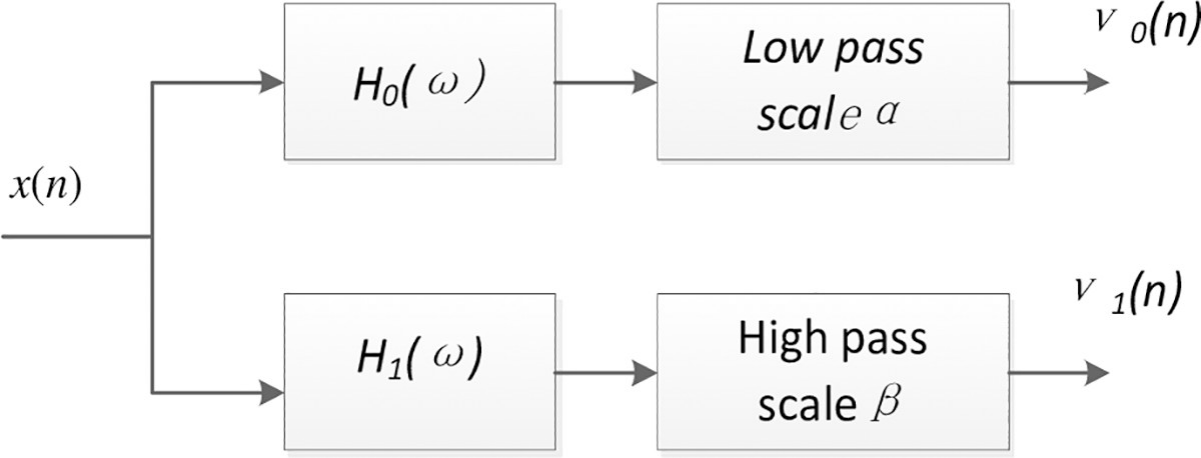
Decomposed filter bank of resonance-based sparse decomposition. A test signal could be decomposed into two components with high-pass(low-pass) scale factor by TQWT.

Wavelet base function libraries which have the characteristics of high and low Q and the corresponding transformation coefficient can be obtained by Tunable Q-Factor Wavelet Transform (TQWT), TQWT decomposes a signal in an iterative manner, which can be seen in Fig 2.

Where, *β* is a high-pass scale factor, $\beta =\frac{2}{Q+1}$; *α* is a low-pass scale factor, $\alpha =1-\frac{\beta }{r}$, *r* indicates redundancy. *v*_0_(*n*) and *v*_1_(*n*) denote the subband signal. The formula for calculating the number of decomposition layers *L* is:

$$L_{\max}\leq \frac{\mathrm{lg}(\beta \times N/8)}{\mathrm{lg}(1/\alpha )}$$ (2)

Where, *N* is the signal scale. The objective function of the ‘morphological component analysis’ (MCA) is set as follows:

$$J(W_{1},W_{2})=∥x-S_{1}W_{1}-S_{2}W_{2}{∥}_{2}^{2}+\sum_{m=1}^{j_{1}+1}\lambda_{1,m}∥w_{1,m}{∥}_{1}+\sum_{n=1}^{j_{2}+1}\lambda_{2,n}∥w_{2,n}{∥}_{1}$$ (3)

Where, *W*_1_, *W*_2_ are the transformation coefficients of signal *x*_1_ and *x*_2_ under frame *S*_1_, *S*_2_ respectively; *m* and *n* are the number of high and low resonance subbands respectively, *λ*_1,*m*_ (*λ*_2,*n*_) are the regularization parameter of the *m*-th (*n*-th) component of the high(low) resonance component, Split-augmented Lagrangian search algorithm is used to updates the transformation coefficients *W*_1_ and *W*_2_ iteratively to minimize the objective function *J*, the corresponding transformation coefficients of high and low resonance component are $W_{1}^{*}$ and $W_{2}^{*}$ respectively, then the estimated values of high and low resonance component are:

$${\hat{x}}_{1}=S_{1}W_{1}^{*},\;{\hat{x}}_{2}=S_{2}W_{2}^{*}$$ (4)

### 2. Variational modal decomposition

VMD is an algorithm that could decompose the input signal x into specified number (K)of quasi-orthogonal band-limited intrinsic mode functions (BLIMFs) *μ*_*k*_. which have unknown but separable spectral bands [25–26]. The fundamental principle of VMD can be expressed as the solving of a constrained variational problem:

$$\underset{\left\{u_{k_{1}}\},\{\omega_{k_{1}}\right\}}{\min}\left\{\sum_{k_{1}}^{K}{\left\|\partial_{t}[(\delta (t)+\frac{j}{\pi t})*\mu_{k}{}_{{}_{1}}(t)]e^{-j\omega k_{1}t}\right\|}_{2}^{2}\right\}$$ (5)

$$s.t\;\sum_{k_{1}}^{K}u_{k_{1}}=f$$

Where *f* is the original signal, *μ*_*k*1_ is the *k*_1_ th intrinsic mode function(IMF) component and *δ*(*t*) denotes pulse signal; *j* is the imaginary unit, $\omega_{k_{1}}$ denotes the center frequency of the *k*_1_ th IMF component; ∥•∥_2_ is 2-norm.

The penalty parameter *α* and LaGrangian multiplier *λ*(*t*) are introduced to solve the above-constrained issue:

$$L(\{u_{k_{1}}\},\{\omega_{k_{1}}\},\lambda (t)=\alpha \sum_{k_{1}=1}^{K}{\left\|\partial t[\delta (t)+\frac{j}{\pi t})*u_{k_{1}}(t)]e^{-j\omega_{k_{1}}t}\right\|}_{2}^{2}+{\left\|f(t)-\sum_{k_{1}}u_{k_{1}}(t)\right\|}_{2}^{2}+<\lambda (t),f(t)-\sum_{k_{1}=1}^{K}u_{k_{1}}(t)$$ (6)

$u_{k_{1}}$ indicates IMFs, $\omega_{k_{1}}$ are corresponding center frequencies. the LaGrangian multiplier *λ*, are subsequently updated as:

$${\hat{u}}_{k_{1}}^{n+1}(\omega )=\frac{\hat{f}(\omega )-\sum_{i\neq k_{1}}{\hat{u}}_{i}(\omega )+\frac{\hat{\lambda }(\omega )}{2}}{1+2\alpha {\left(\omega -\omega_{k_{1}}\right)}^{2}}$$ (7)

$$\omega_{k_{1}}^{n+1}=\frac{{\int_{0}^{\infty }\omega \left|{\hat{u}}_{k_{1}}(\omega )\right|}^{2}d\omega }{{\int_{0}^{\infty }\left|u_{k_{1}}(\omega )\right|}^{2}d\omega }$$ (8)

$$\lambda^{n+1}(\omega )=\lambda^{n}+\vartheta [f(\omega )-\sum_{k_{1}=1}^{K}u_{k_{1}}^{n+1}]$$ (9)

The above update process is implemented repeatedly until the following convergence criterion is satisfied:

$$\sum_{k_{1}=1}^{K}\frac{∥u_{k_{1}}^{n+1}-u_{k_{1}}^{n}{∥}_{2}^{2}}{∥u_{k_{1}}^{n}{∥}_{2}^{2}}<\varepsilon$$ (10)

Where *ε* is set as 10^−6^.

## Resonance-based sparse adaptive variational mode decomposition

### 1. Main idea

Frequency analysis and filtering are basic methods for signal processing. but they cannot be effectively applied to all kinds of signals; they are useful for signals that are essentially oscillating or periodic.

VMD functions as a Wiener filter in the Fourier domain as mentioned earlier, But it could not analyze signals which have overlapping spectra, this signal usually appear in wideband non-stationary signals. To prove the limitation, a nonstationary signal which has two components is introduced:

$$\begin{array}{l} s(t)=s_{1}+s_{2}\\ =e^{-0.3t}\times \cos(2\pi (60t+120t^{2}))\\ +e^{-0.5t}\times \cos(2\pi (120t+240t^{2})) \end{array}$$ (11)

**Fig 3:**
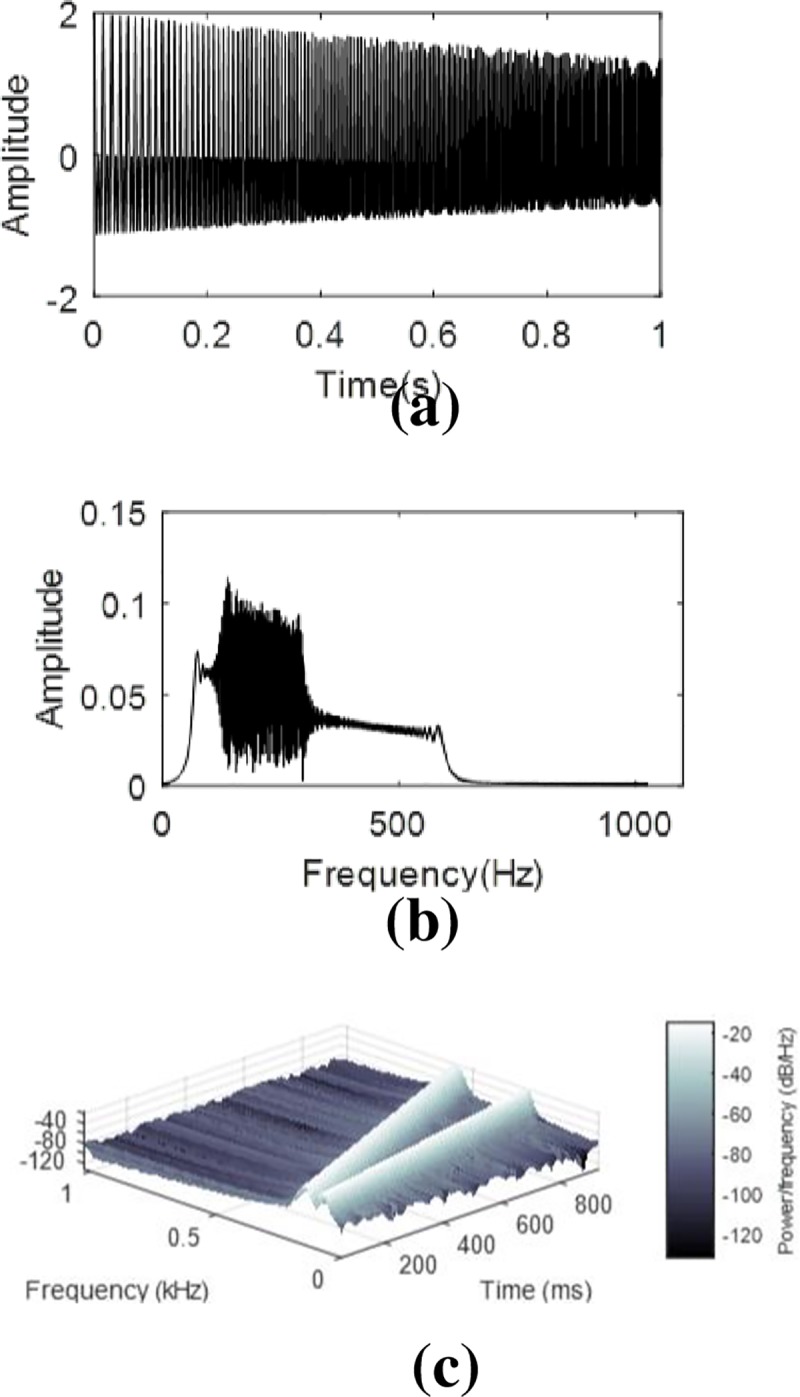
The test signal. (a) Waveform. (b) Frequency spectrum. (c) TFR with the STFT. The instantaneous frequency of *s*_1_ and *s*_2_ are *f*_1_(*t*) = 50+200*t*, *f*_2_(*t*) = 120+480*t*.

Fig 3 shows the information about this signal.

**Fig 4:**
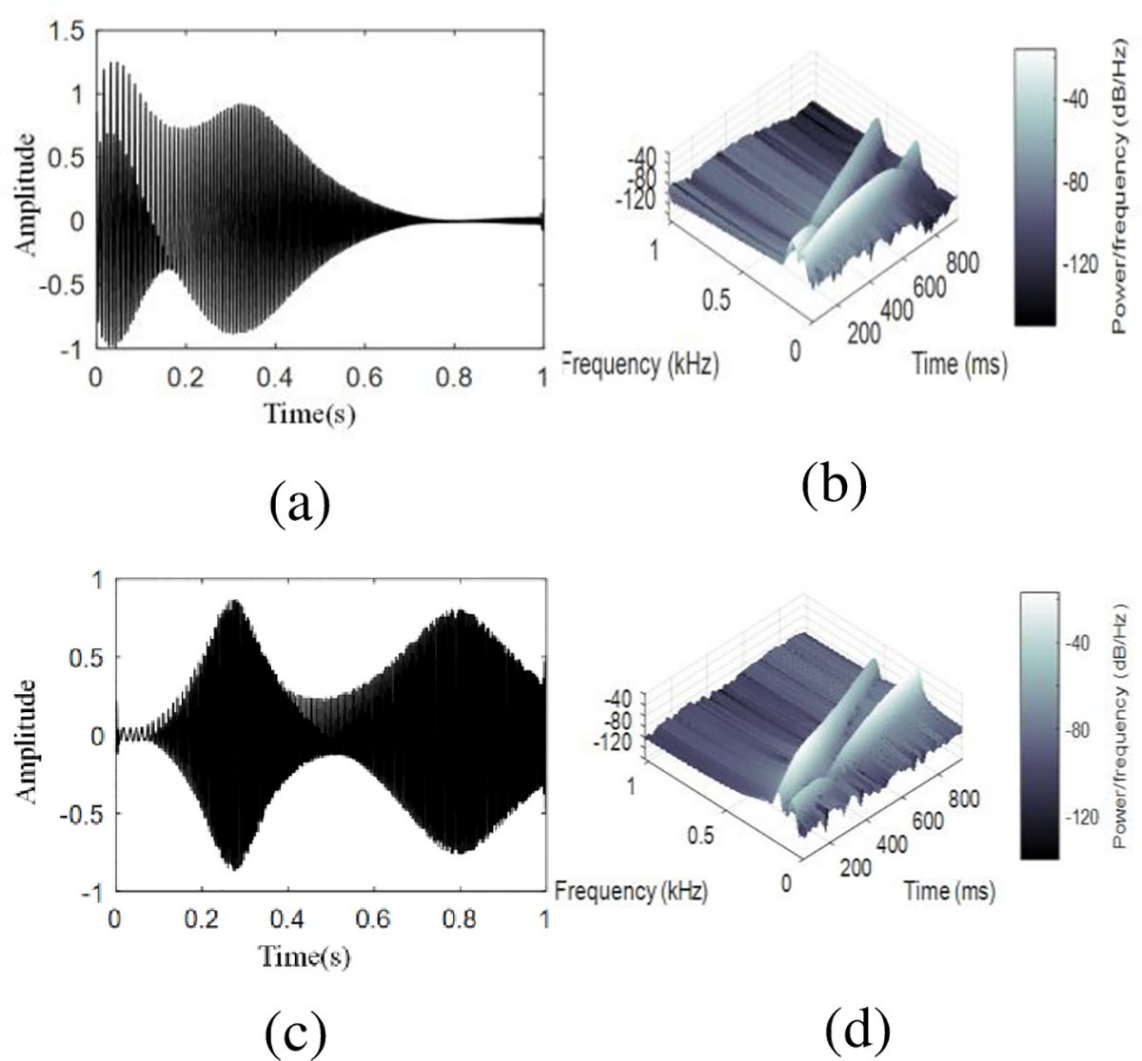
The VMD decomposition results of the signal in (11) (a) waveform of IMF1 (b) TFR with the STFT of IMF1. (c) waveform of IMF2 (b) TFR with the STFT of IMF2.The waveform of IMF1 and IMF2 show that they both contain two components. TFRs shows that they have two frequency components.

The Fig 3(B) indicates that the signal has a wideband spectrum, and the Fig 3(C) shows the instantaneous frequency of the two components vary linearly. The decomposition results of VMD are shown in Fig 4.

From Fig 4 it can be observed that s1 and s2 are both divided into two parts (as seen in Fig 3(B) and 3(D)). VMD cannot decompose a signal with overlapping spectra.

In fact, the signals obtained by planetary gearboxes are often composed of continuous oscillations and transient phenomena, which are difficult to calculate using linear methods. The spectrum of some broadband signals has overlapping components and cannot be separated in the frequency domain, but can be separated in another domain.

A signal can be viewed as the sum of a ‘high-resonance’ and a ‘low-resonance’ component. The high-resonance components are consisted of multiple simultaneous sustained oscillations and the low-resonance components are consisted of non-oscillatory transients of unspecified shape and duration.

**Fig 5:**
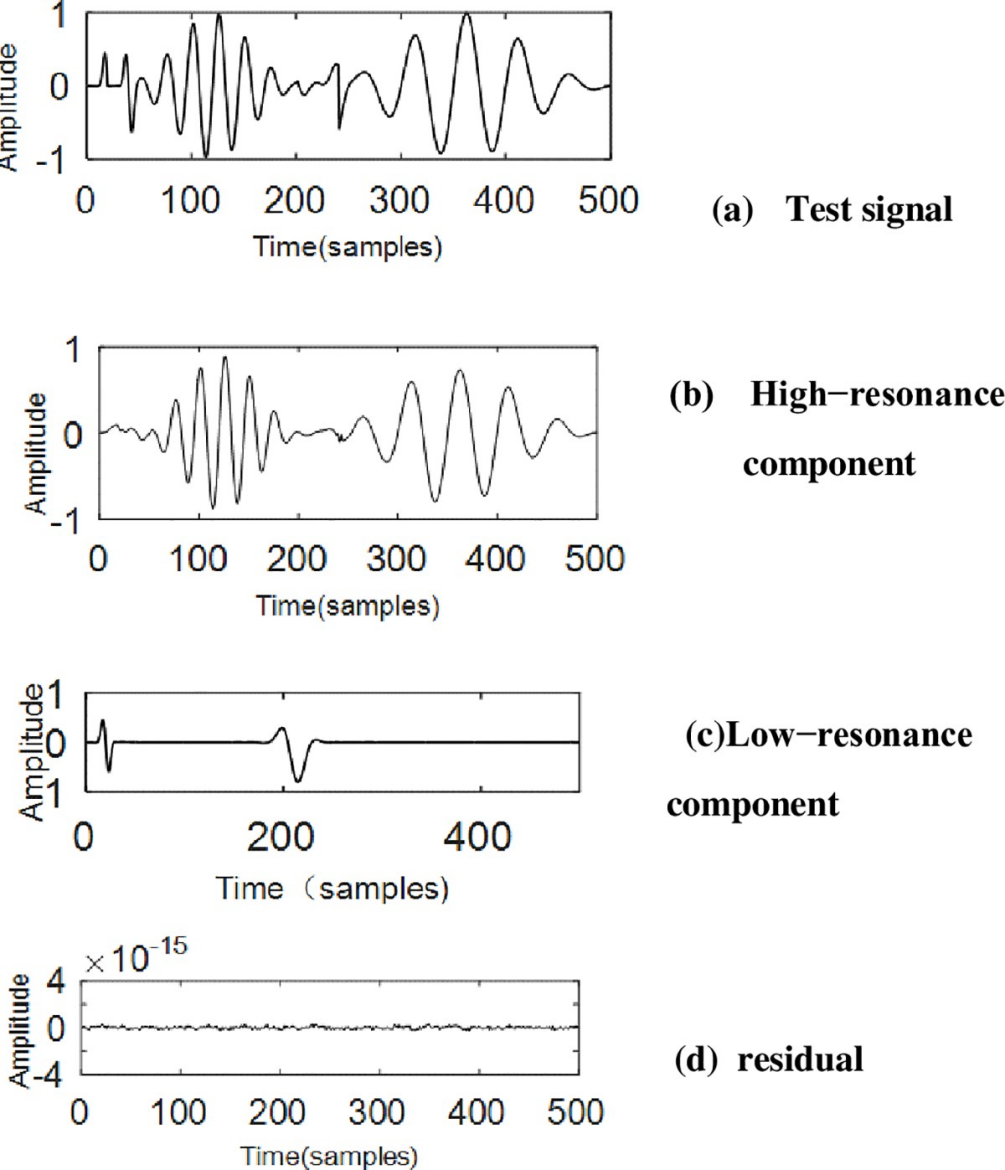
The results of synthetic test signal in Fig 1 by resonance-based decomposition. Fig 5(A) shows the test signal composed of four impulse signals in Fig 1, Fig 5(B) shows the high resonance component after decomposition, and Fig 5(C) shows the low resonance component after decomposition, showing no component overlap. Fig 5(D) shows the residual signal after decomposition.

Resonance-based signal decomposition should be able to separate the pulses when their resonance is different. The resonance-based signal decomposition algorithm is applied to the synthetic test signal to illustrate the effects of its decomposition. The test signal consists of 4 pulses of two levels of resonance in Fig 1 (Fig 5 shows the results of synthetic test signal in Fig 1 by resonance-based decomposition). Therefore, this paper makes use of the characteristics of TQWT and VMD, proposed a new method which is mainly aimed at multi-component chirp signals (MLFMs). MLFMs are a kind of non-stationary signals that often appear in the vibration signals of radar, sonar, communication, and rotating machinery.

**Fig 6:**
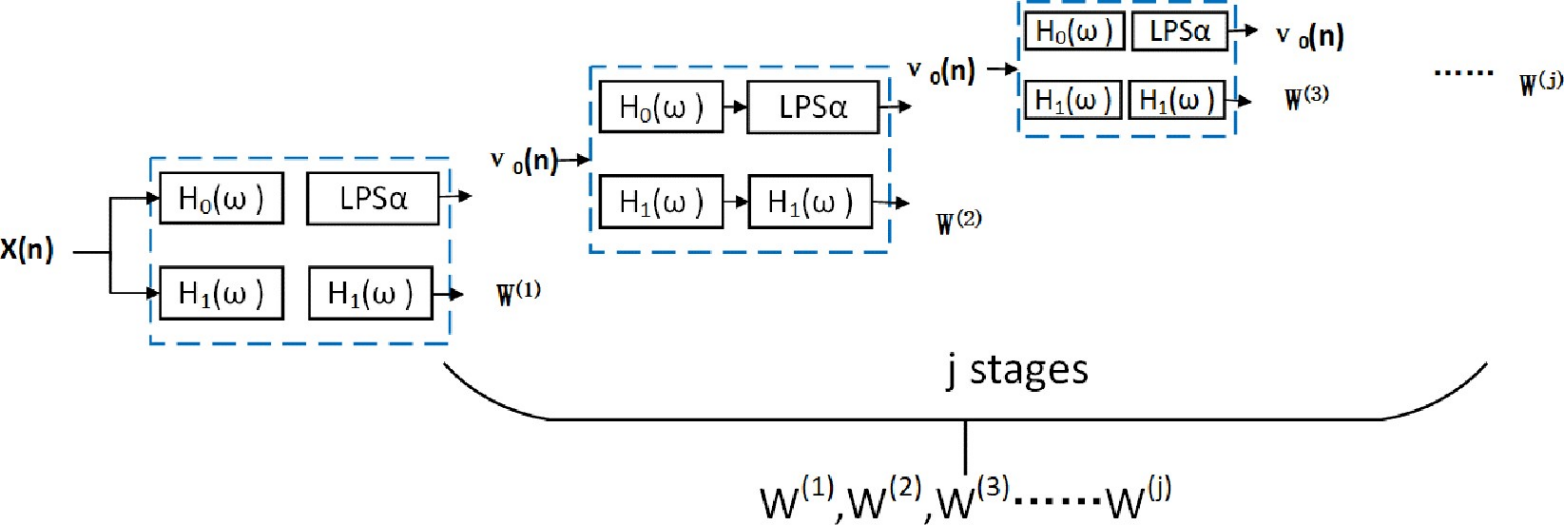
The components of TQWT. TQWT is composed of j—layer two-channel decompose filter banks.

Firstly, Select the initial value of Q_H_,Q_L_,the MLFMs is processed by TQWT, Fig 6 shows the general two-channel decompose filter banks. *H*_0_(*ω*) represents the frequency response function decomposition filter. *α* and *β* are low and high scale factor.

Secondly, The signal is separated by ‘morphological component analysis’ (MCA), energy analysis is conducted to select the components which contain more energy in the process of MCA, and High resonance signal x_H_ and the Low resonance signal x_l_ is reconstructed by them.

Thirdly, the reconstructed signal x_H_, x_L_ is decomposed by Adaptive variational mode decomposition (AVMD),the IMFs with the Maximum kurtosis was selected as x_1_,x_2._

Fourthly, iterative over above process until minimizing the Objective function. The objective function is:

$$J(Q_{H},Q_{L})=∥x-x_{1}-x_{2}{∥}_{2}^{2}+Jaccard(x_{1},x_{2})$$ (12)

The binomial norm of the first term is used to keep the residual components to a minimum. Meanwhile, Jaccard similarity coefficient is introduced to measure the similarity of x_1_ and x_2_.the Q_H_ and Q_L_ is selected to keep the sum of the residual components and Jaccard(x_1_,x_2_) to a minimum.

**Fig 7:**
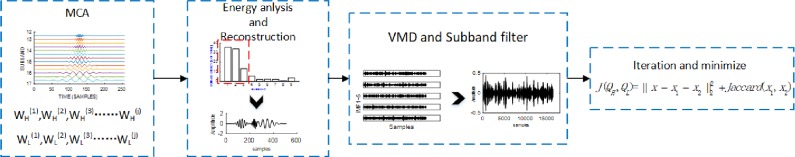
Main idea of the proposed method RSAVMD. In order to select the appropriate Q_H_ and Q_L,_ the signal (after TQWT)was processed by MCA firstly, energy analysis was conducted for each component, the component with more energy was reconstructed, AVMD analysis was conducted for the reconstructed component, and the above process was iterated until minimizing the objective function. At this point, envelope spectrum analysis was performed for AVMD results with high resonance components.

Finally, the envelope of the high components x_1_ is analyzed to extract fault feature frequency. Fig 7 shows the main idea of proposed method.

### 2. The adaptability of VMD

In recent years, the adaptive process of VMD has attracted much attention. Literature [27] uses the mutual information of decomposition margin and original signal to judge whether the original signal is completely decomposed. Literature [28] calculates the difference of center frequencies of different components, and when the difference of frequency centers is relatively close, it is deemed that redundant components appear. However, the low similarity between the decomposition margin and the original signal does not mean that the decomposition is complete. On the contrary, excessive decomposition may occur. The difference between the center frequencies still needs to be chosen manually and empirically.

**Fig 8:**
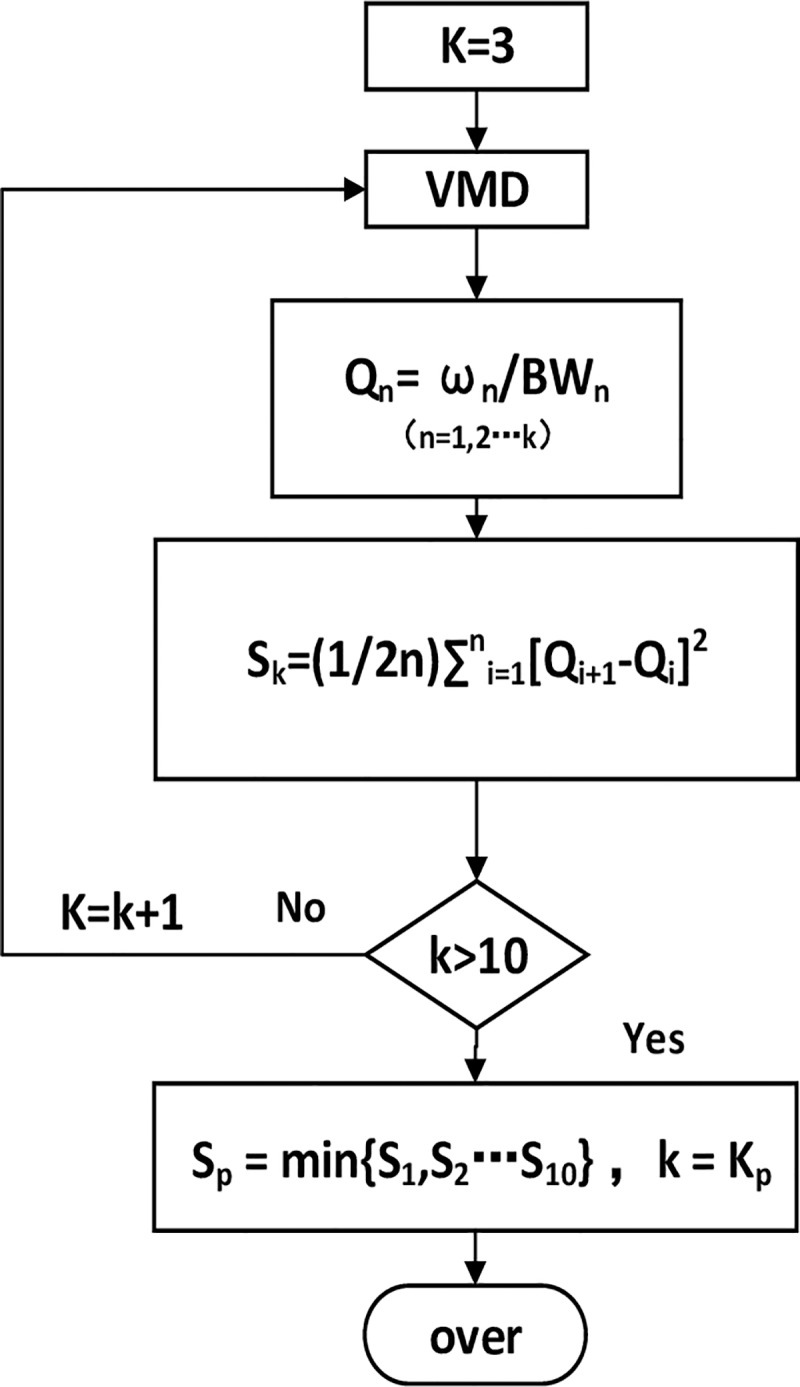
The process of adaptive VMD. Different k values are selected until the variation S_k_ of Q of different IMFs is minimized.

Since the VMD ensures that the sum of the aggregation bandwidths of each component is the minimum, and each decomposed variational modal component has a specific sparse attribute, the ratio of the center frequency to the bandwidth of different components which named the quality factor (Q)presents a regular distribution. If the Q value of a component suddenly increases or decreases, it is because the center frequency is too large (bandwidth is too small) or too small (bandwidth is too large), which will lead to the K value being too large or small. The variation *s* is used to measure the stability of quality factor Q. When *s* is the minimum, the distribution of Q of each component is stable and the center frequency and bandwidth are optimized. The process of adaptive VMD is showed in Fig 8.

1. Set the initial value k = 2 of the decomposition number of VMD, the signal was decomposed into k IMFs by VMD.
2. Calculate the Q_n_ (n = 1,2…k) of different IMFs.
3. The variation *S*_*k*_ of the Q_n_ was calculated. Let K = K+1, repeat step 1), until K value is equal to 10 (when the decomposition number is too large, false components will appear. According to experience, the highest decomposition number selected in this paper is 10).
4. The number of components corresponding to the minimum value of the variation S_k_ is taken as the number of optimal components named K_p_.

## Experimental analysis of planetary gear box

### 1. Experimental facility and manufacturing planetary gearbox failure

**Fig 9:**
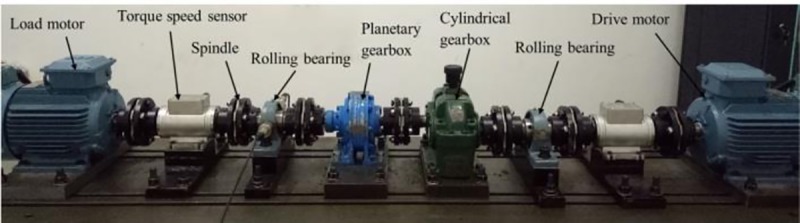
Wind power transmission system structure simulation test bench. The drive motor simulates the wind wheel torque input and connects the load motor after the secondary gear transmission. In order to simulate the time-varying features of wind turbine speed, a frequency converter is used to control the motor speed. Various types faults of gear box and bearing were prepared by manual machining of defects.

The schematic diagram of the wind power drive train simulation test bench is shown in Fig 9.

**Fig 10:**
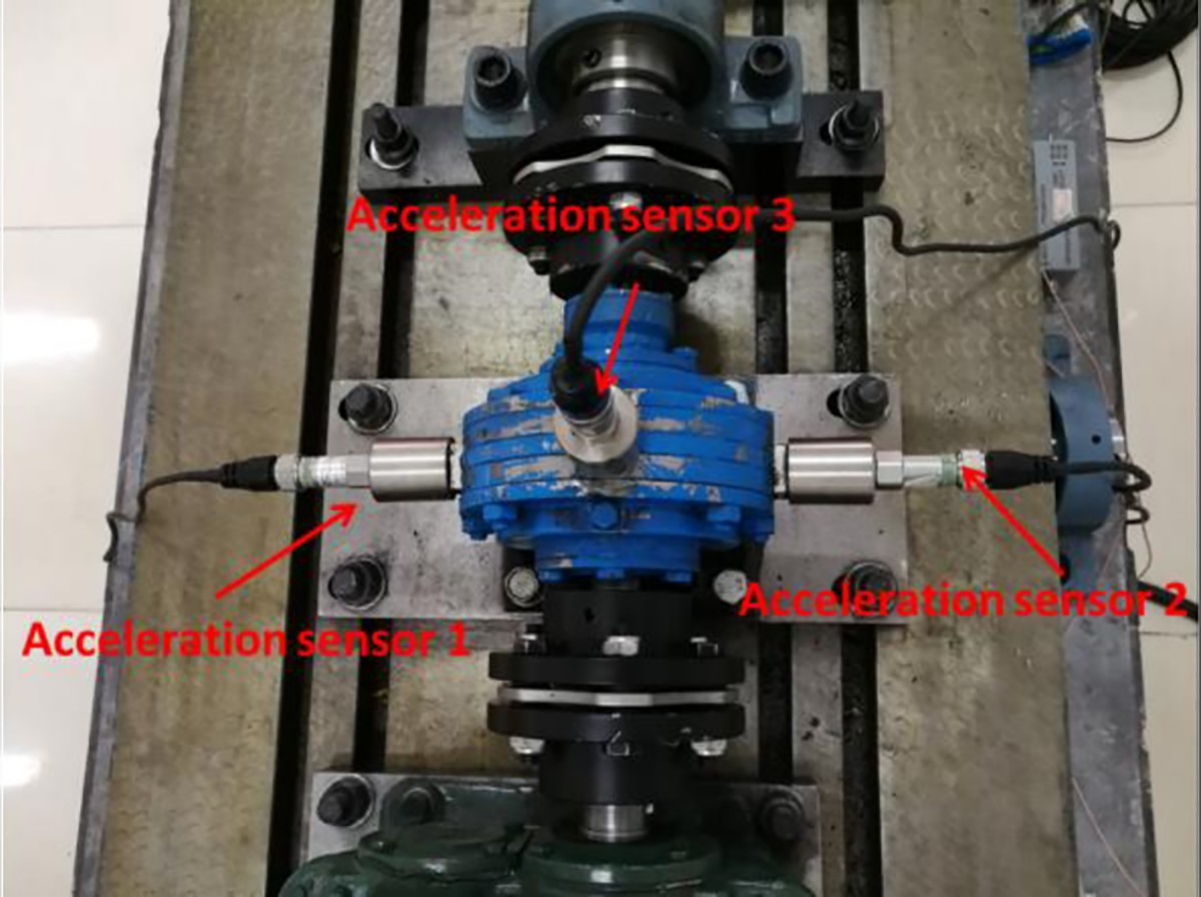
The layout of the planet gear box vibration sensor. Vibration sensors are mounted on both sides of the planetary gear housing.

The breaking fault of planetary gear of planetary gearbox studied in this paper is prepared by the EDM technology to smooth the 0.5 times tooth length of a certain tooth on the planetary gear. The arrangement of sensor measuring points is shown in Fig 10.

In the fault test of planetary gear box, the parameters of planetary gear box and the parameters of setting working conditions are shown in Table 1.

**Table 1:** Planetary gearbox parameters and the state parameters under the set working condition.

Planetary gearbox parameters		state parameters under the set working condition	
parameters	values	parameters	values
Solar tooth number *z*_*s*_	33	Motor speed *n* (r/min)	500
Number of planetary wheel teeth *z*_*p*_	19	Planetary wheel rotation frequency *f*_*rp*_ (Hz)	8.3
Ring gear teeth *z*_*r*_	72	The rotation frequency of the planetary frame *f*_*rc*_ (Hz)	2.39
Input power *p*_*i*_ (kw)	3	Solar rotation frequency *f*_*ru*_ (Hz)	8.3
The actual speed ratio	1/3.1875	frequency of sampling *f*_*s*_ (Hz)	2048

Under the setting condition, calculate the meshing frequency *f*_*m*_ of the planetary gear box as:

$$f_{m}=f_{rc}Z_{r}=2.39\times 72=172Hz$$ (13)

The feature frequency *f*_*p*_ of planetary wheel failure is:

$$fp=\frac{f_{m}}{z_{p}}=\frac{172}{19}=9Hz$$ (14)

**Fig 11:**
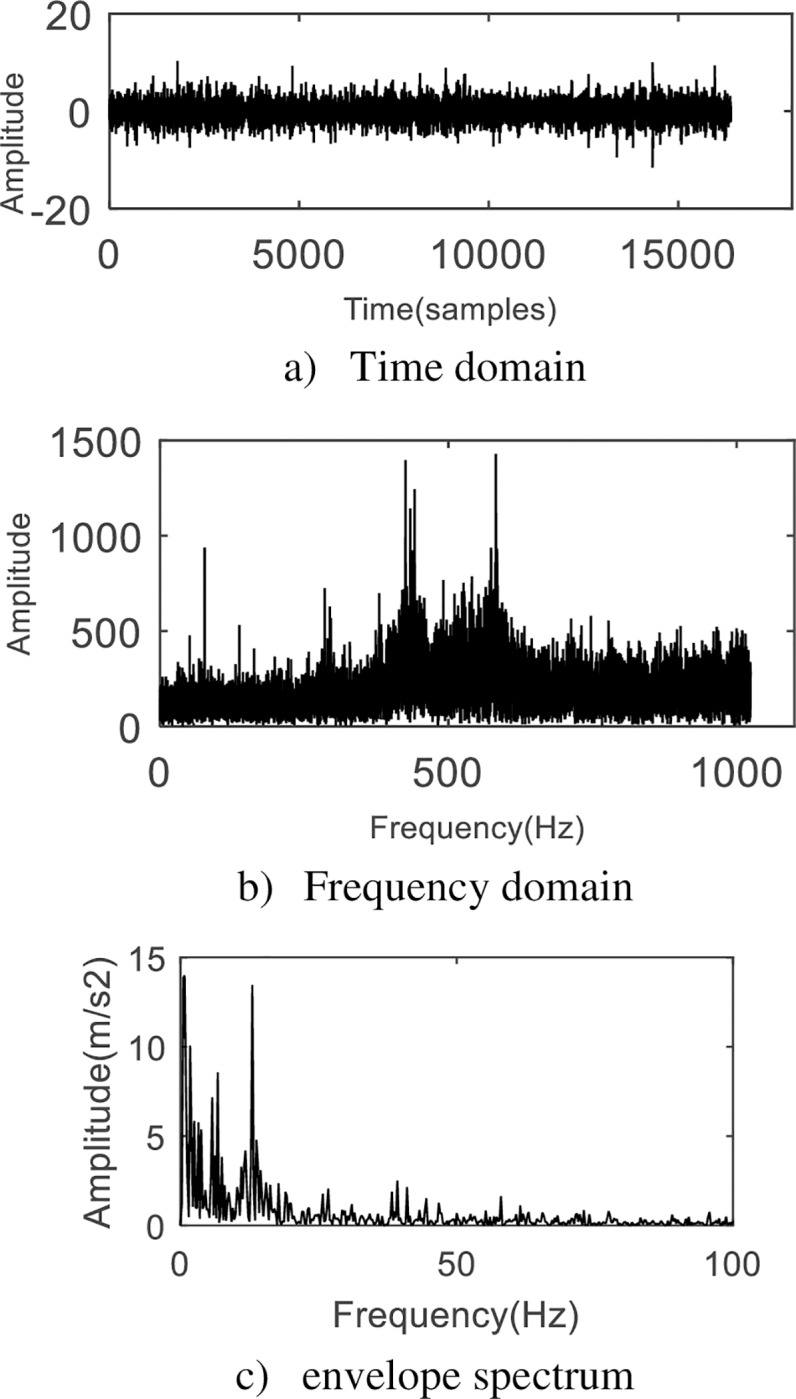
The analysis of original experimental signal. No obvious periodicity could help find fault characteristics.

Acquire vibration signals under the setting conditions, and their time domain and envelope spectrum are shown in Fig 11.

The waveform in Fig 11A) presents cluttered, with no obvious periodicity, and it is impossible to extract valid information. Envelope spectrum of signal in Fig 11C) shows that there are many low frequency components but no obvious peak frequency in the fault state. This is because the test condition contains a lot of noise generated by other components. The fault frequency of the planetary gearbox could be submerged in the noise, and the frequency of fault features of the signal cannot be obtained.

## 2. Planetary gearbox fault diagnosis based on RSAVMD

Analyze the signal by RSAVMD. The number of decomposition K is selected firstly, the Q of components in the case of different decomposition Numbers (only the variation value was listed when k = 3–10 due to the space limitation) was obtained, as shown in Table 2.

**Table 2:** Q values of different components with different K values.

K	Q1	Q2	Q3	Q4	Q5	Q6	Q7	variation
**K = 3**	0.354	0.413	4.443	\	\	\	\	11.020
**K = 4**	0.354	0.413	3.351	3.851	\	\	\	10.500
**K = 5**	0.351	0.414	1.165	3.652	3.361	\	\	10.200
**K = 6**	0.352	0.755	0.43	3.271	4.292	3.350	\	15.340
**K = 7**	0.352	0.760	0.425	1.166	3.270	4.241	3.350	15.880
**K = 8**	\							15.411
**K = 9**	\							22.630
**K = 10**	\							22.950

When K = 5, the variation of Q of different components is the smallest, so the value of K is 5.

**Fig 12:**
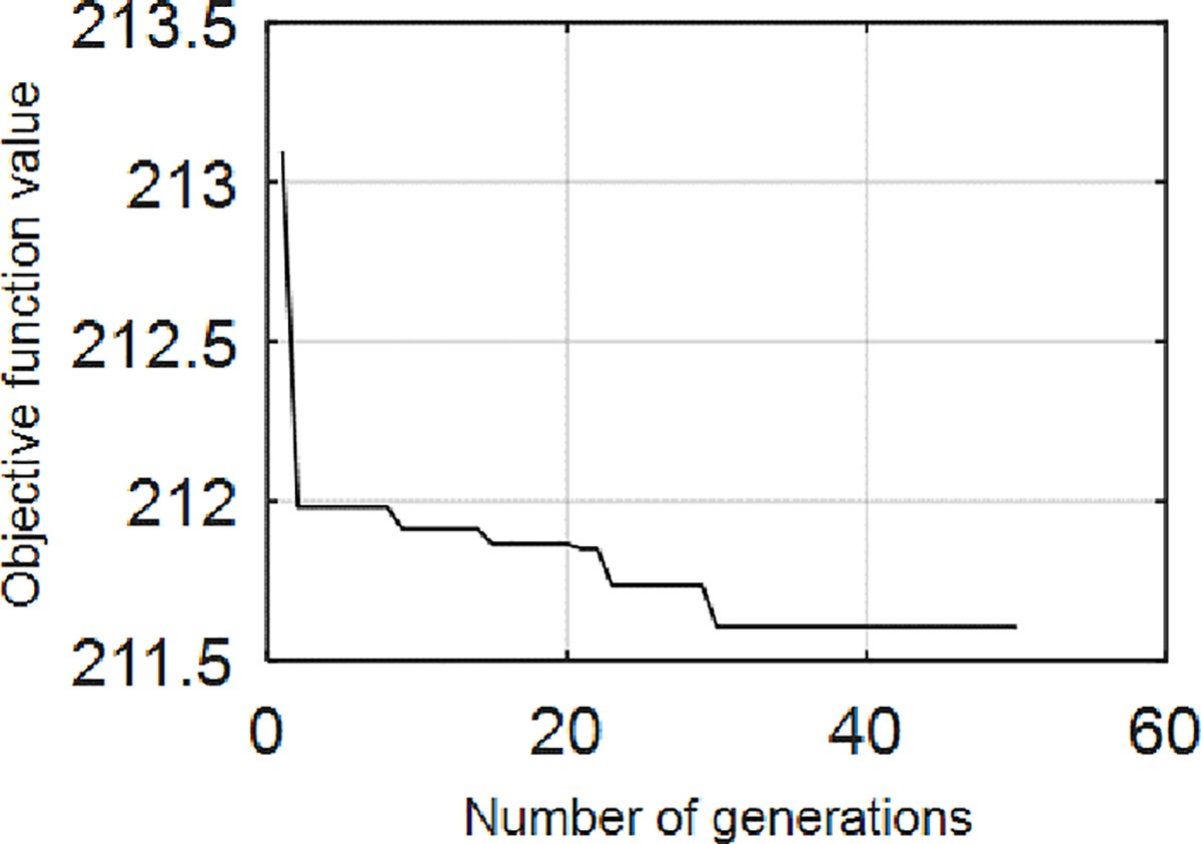
The iteration process of RASVMD. The result of the iteration is that the minimum value of the objective function is 211.7.

The proposed RSAVMD was performed on the experimental signal. And the iterative process was shown as Fig 12.The selected QH and QL are 5.9098 and 1.0683.

**Fig 13:**
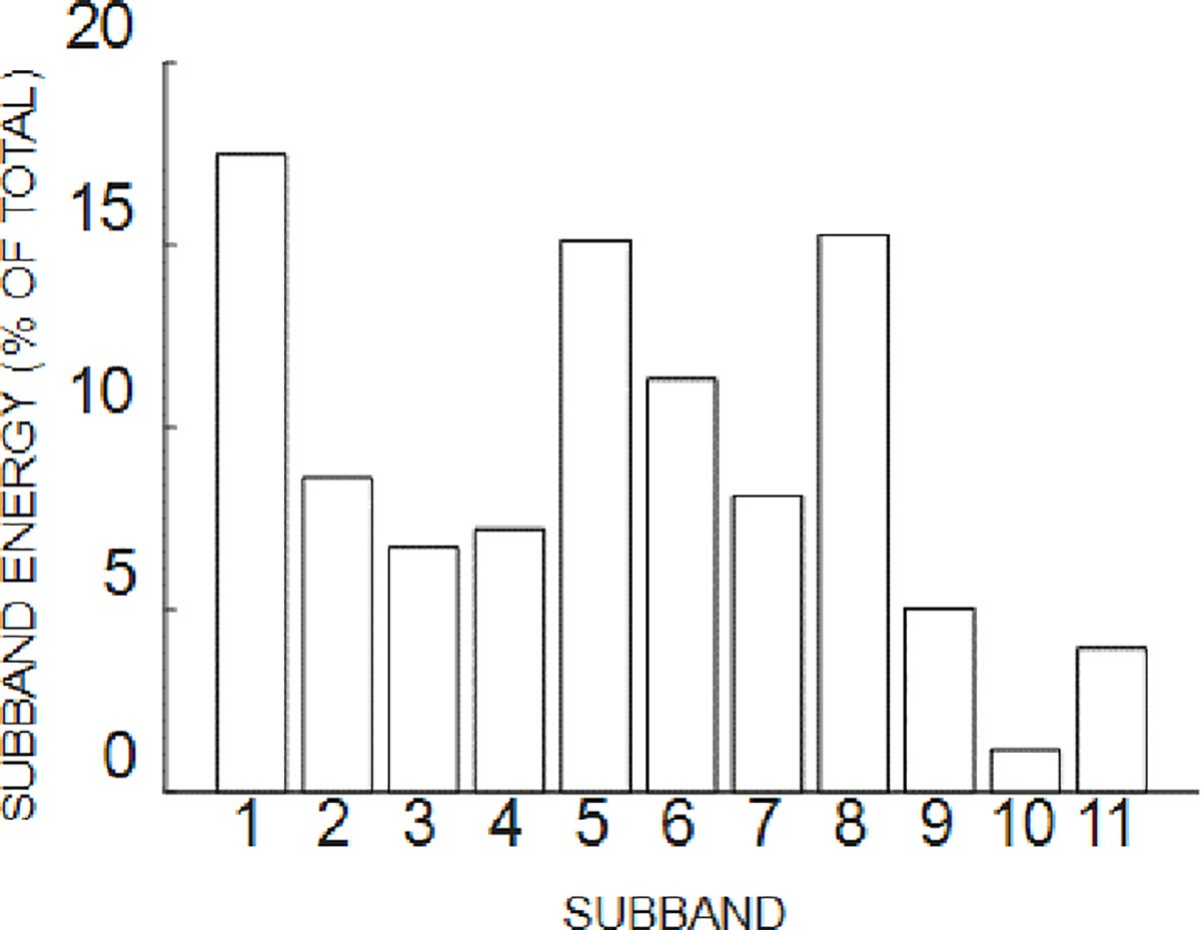
The distribution of the energy of signal HRC. The higher the percentage of energy of per component in the total signal, the higher the bar chart.

The signal is decomposed into high resonance component (HRC) and low resonance component (LRC) with QH and QL. the distribution of the energy of signal HRC is shown in Fig 13.

The signal HRC was reconstructed by 1,2,5,6,7,9,10 components. And then decompose the HRC into 5 components using the VMD and the Relative entropy between these 5 components and the original signal is calculated, as shown in Table 3.

**Table 3:** Relative entropy of each component and the original signal.

components	1	2	3	4	5
**Relative entropy**	**49.6**	**7.28**	**31.72**	**27.12**	**13.01**

**Fig 14:**
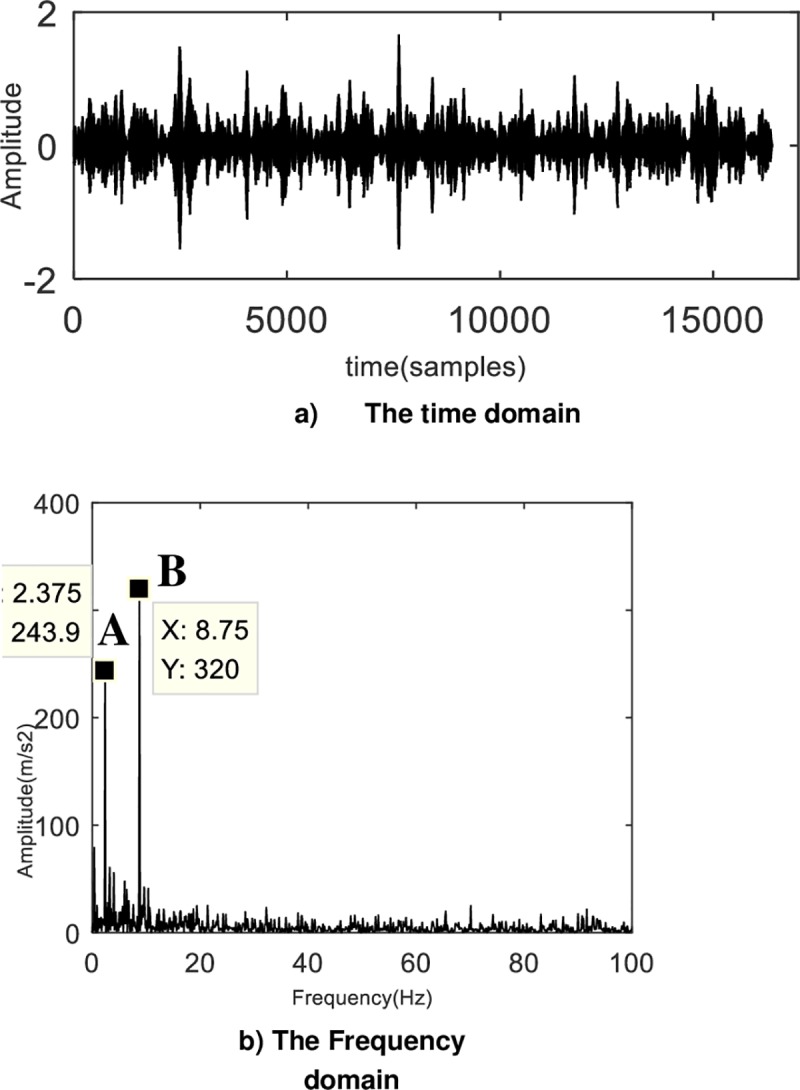
The waveform and envelope spectrum of the fault signal after processing by RASVMD. Comparing Fig 14A) with Fig 11A), the peak amplitude of vibration is further reduced, and the periodic shock of signal waveform is more obvious. Fig 14B) shows the feature frequency compared to Fig 11C), and point A is the rotation frequency of the planet carrier, B is the frequency of fault features of the planetary gearbox.

The component with the smallest Relative entropy is the second component, and the second component is subjected to the envelope spectrum analysis. The time domain and frequency domain analysis of the fault signal obtained by the method of the present invention is shown in Fig 14.

It is proved that the method proposed in this paper can accurately extract the frequency of fault features of planetary gearbox.

### 3. Comparison of two methods of VMD and RSAVMD

**Fig 15:**
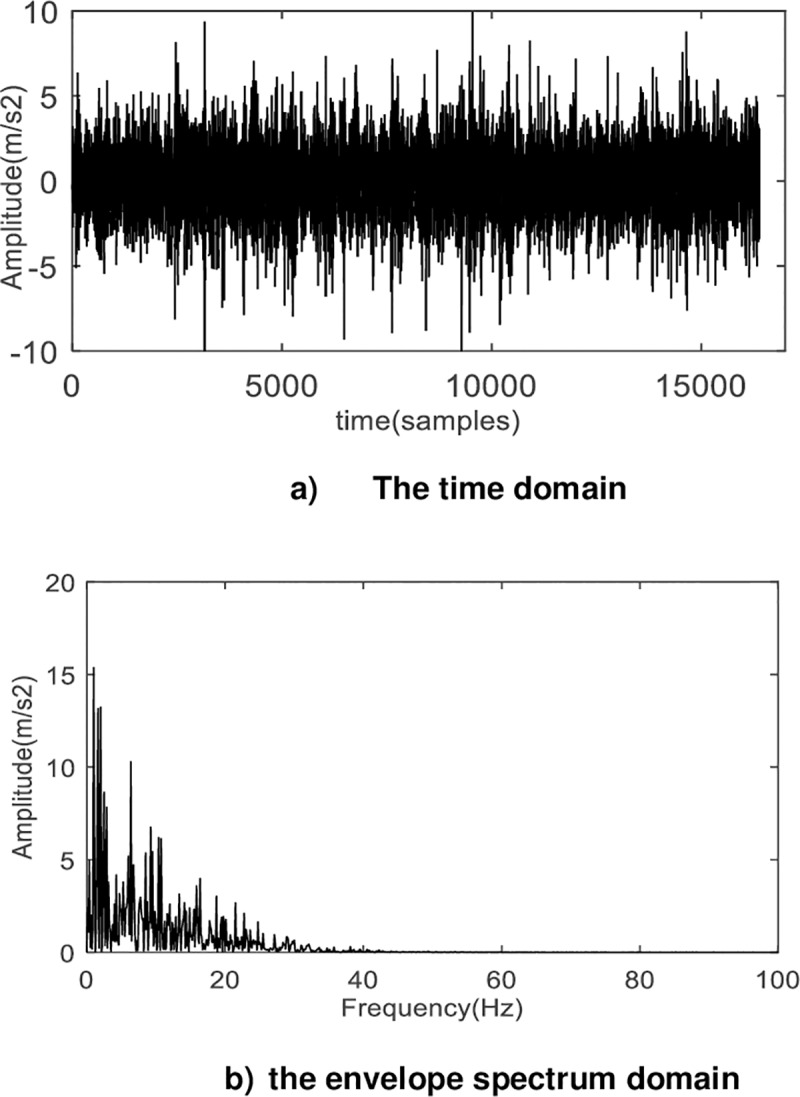
The analysis of original experimental signal by VMD a) The time domain b) The frequency domain.

In order to prove the effectiveness of the method proposed in this paper further, the effects of VMD and RASVMD are compared, the VMD was applied to experimental signals. Fig 15 shows the waveform and envelope spectrum after processing by VMD.

As can be seen from the Fig 15, the waveform has no obvious periodicity, so the fault characteristics cannot be extracted. Meanwhile, there are many low-frequency components in the envelope spectrum, so the fault characteristic frequency could not be extracted. Clearly. Thus, RASVMD is superior to VMD. At the same time, the Q-factor of each unit only needs to be selected once, so the selection process does not include the running time of RSAVMD, which is 19.721991, and the running time of VMD is 19.591333.The running times of the two methods are similar.

### 4. Comparison of two kinds of adaptive VMD

**Fig 16:**
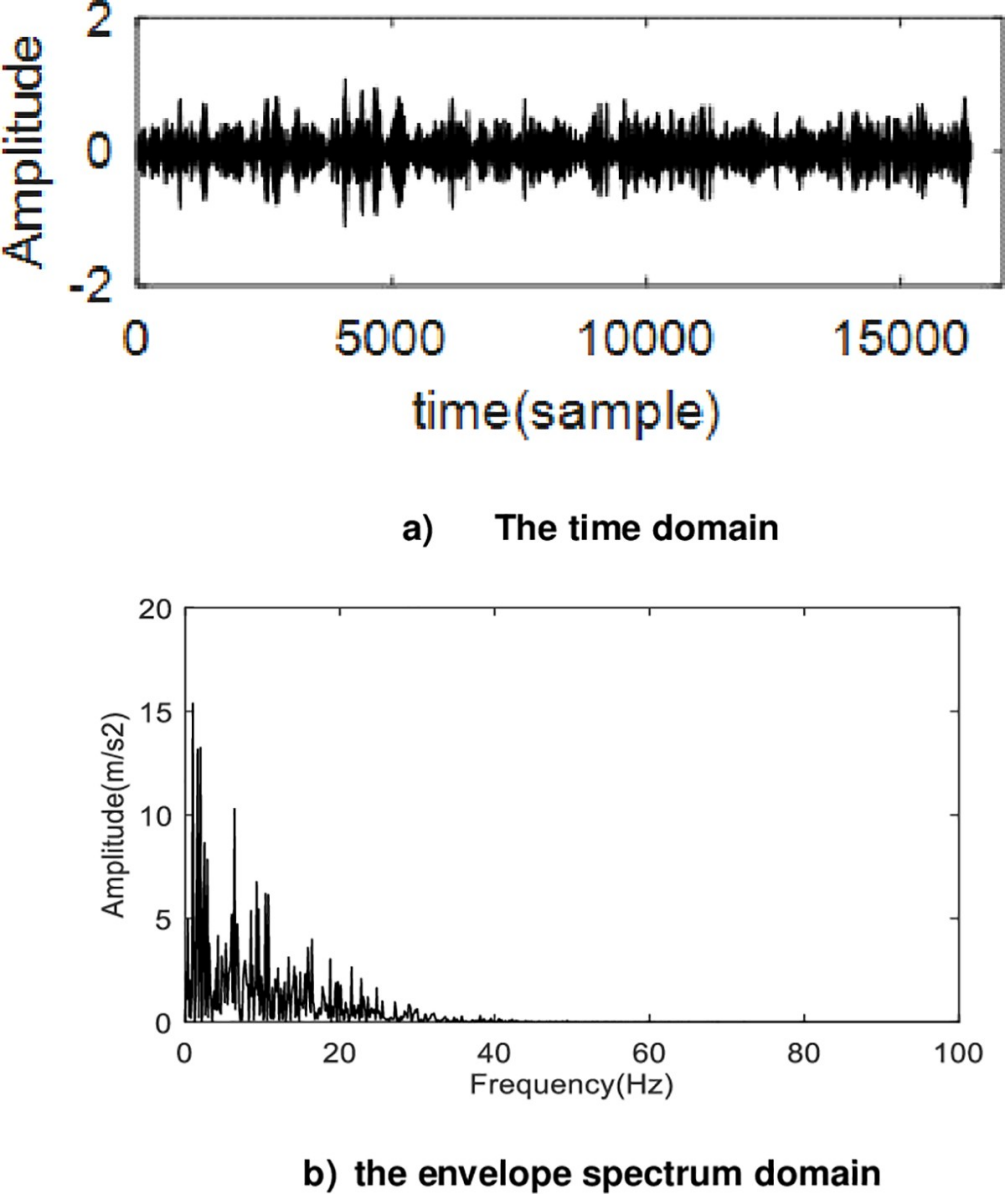
The waveform and envelope spectrum of fault signals after the decomposition of VMD whose decomposition number is determined by method in literature [27]. The envelope spectrum of this method could not display characteristic frequency.

The method used in literature [27] is adopted to determine the number of VMD decomposition, so as to extract the frequency of fault features of planetary gear box. Firstly, the central frequency of each component under different decomposition Numbers is calculated. When K = 6, the difference between the central frequency of different components is small, so the number K is determined to be 6. The Relative entropy between the 6 components and the original signal was calculated, and the component with the smallest Relative entropy was taken, and then the envelope spectrum analysis was conducted to obtain Fig 16.

As shown in Fig 16, the waveform of the signal shows that the peak of the waveform decreases but the periodicity is not much more obvious. the envelope spectrum of this method can not display characteristic frequency. Therefore, the number of components determined by the method in this paper is more accurate. At the same time, this comparison also shows that the original VMD cannot accurately extract the fault characteristic frequency of the planetary gearbox.

## Conclusions

The planetary gearbox vibration signal has strong background noise, complex sideband, and obvious amplitude and frequency modulation. It is difficult to extract the feature frequency of fault by conventional methods. This paper proposes RSAVMD to solve the problem that the weak robustness of VMD and the number of decompositions cannot be adaptive. The conclusions drawn are as follows:

1) During the analysis of experimental signals, the decomposition number of VMD was determined by using the method based on center frequency in literature [11] and Q respectively. The experiment showed that the decomposition number determined by the method based on center frequency resulted in the loss of part of the characteristic frequency(the rotation frequency of the planetary frame), while the extracted fault characteristic frequency was not obvious. The adaptive VMD method based on quality factor can decompose the rotation frequency and fault characteristic frequency accurately.

2) The comparison of this paper also shows that the RSAVMD solved the problem of VMD. The essence of VMD is multiple adaptive wiener filter banks, but the use of filter must be used in frequency domain, VMD could not deal with the signal whose spectrum has overlap. However, Signal components with similar center frequency but different resonance properties can be separated effectively by RSAVMD and wideband signal which is not to do with planetary gear fault signals can be eliminated. At the same time, the decomposition number of VMD needs to be selected artificially, and RSAVMD realizes the adaptive selection of decomposition number. In the envelope spectrum, the fault characteristic frequency of the broken tooth of the planetary wheel is presented clearly. It is helpful for engineering diagnosis.

## Supporting information

S1 Data (RAR)
